# Population indices measuring health outcomes: A scoping review

**DOI:** 10.7189/jogh.09.010405

**Published:** 2019-06

**Authors:** Khalid Ashraf, Chirk Jenn Ng, Chin Hai Teo, Kim Leng Goh

**Affiliations:** 1 Department of Primary Care Medicine, Faculty of Medicine, University of Malaya, Kuala Lumpur, Malaysia; 2 Department of Applied Statistics, Faculty of Economics and Administration, University of Malaya, Kuala Lumpur, Malaysia

## Abstract

**Background:**

Population health indices such as disability adjusted life years (DALY) and quality adjusted life years (QALY) are often used in an effort to measure health of populations and identify areas of concern that require interventions. There has been an increase of number of population health indices since the last review published more than a decade ago. Therefore, this study aims to provide an overview of existing population health indices and examine the methods used to develop them.

**Methods:**

The search was conducted across three databases: PubMed, CINAHL and Emerald using four key concepts: ‘health’, ‘index’, ‘context’, ‘develop’, which was supplemented with Google searching and reference scanning. A researcher screened the titles, abstracts and subsequently full texts and confirmed the findings with the research team at each stage. Data charting was performed according to the included publications and identified indices. The collation was performed by describing the indices and made observation on its development method using *a priori* framework consist of four processes: underpinning theory, model or framework; data selection and processing; formation of index; testing of index.

**Results:**

Twenty-six publications describing population health indices were included, and 27 indices were identified. These indices covered the following health topics: overall health outcomes (n = 15), outcomes for specific health topics (n = 4), diseases outcome (n = 6), assist health resource allocation for priority minority subgroup or geographic area (n = 4), quality of health or health care (n = 2). Twenty-one indices measure health for general populations while six measure defined subpopulations. Fourteen of the indices reported at least one of the development processes according to the *a priori* framework: underpinning theory, model or framework (n = 7); data selection and processing (n = 8); formation of index (n = 12); testing of index (n = 9).

**Conclusions:**

Few population health indices measure specific health topics or health of specific sub-population. There is also a lack of usage of theories, models or framework in developing these indices. Efforts to develop a guideline is proposed on how population health indices can be developed systematically and rigorously to ensure validity and comprehensive assessment of the indices.

Population health is a field of research that has been growing rapidly in recent years. Population health approach aims to improve health of the entire population and reduce health inequities within population. It is a broad field ranging from the study of diverse set of health outcomes and determinants to the discourse of health policy intervention [1]. Kingdon argued that the authorities attend to various problems at a time, and this in turn makes some problems prioritized and others ignored. He added, one of the facilitators that captures the attention of the authorities are indicators, which are used in two major ways: to assess the magnitude of a problem and to become aware of changes in the problem [2]. In the context of health policy discourse, health indicators are excellent means of promoting statistical comparability within and among health care systems. Health indicators are statistics selected to represent relevant characteristics of population health because of their power to summarize, or to serve as indirect or proxy measures for information that is lacking [3].

In recent years, researchers have combined population health indicators into an index to gain a wider perspective for their study. An index is defined by the Organisation for Economic Co-operation and Development (OECD) as “a composite indicator that is formed when individual indicators are compiled into a single index, on the basis of an underlying model of the multi-dimensional concept that is being measured” [4]. A population health index therefore provides a summary measure of a certain health characteristic at the population level. For instance, disability adjusted life years (DALY), which combines number of years lost due to premature death and number of years lost due to disability burden, was developed as a measure of the burden of disease. DALY as a single unit enable a multidimensional assessment of health outcome to be considered in comparison with a unidimensional unit for resource allocation, in unit of dollar for example. It provides a comparable measure of output for the design of an intervention or programme for different health problems, which helps to shift the relative weight of political decisions to evidence-based decision making in health resource allocation [5].

There are many population indices that were developed around the globe. Bandura’s survey listed 178 indices, while Yang’s inventory captures 101 indices [6,7]. However, these compilations of population indices include topics beyond health. In addition, both did not use any systematic searching approach for their compilation. Hence, the comprehensiveness of the population indices listing is questionable. In 2004, Kaltenthaler et al published a systematic review to identify articles describing population health indices and their development [8]. This systematic review, which was published before the PRISMA statement [9], did not describe an explicit reproducible method [10], particularly a clearly defined eligibility criteria and search strategy. Nevertheless, Kaltenthaler et al argued that a review on population health indices is beneficial because indices may facilitate the consideration of allocating and targeting health resources based on health needs from a multidimensional perspective.

Given the range of methodological variants in developing an index [6-8], an overview of the methodological approaches is essential. This will benefit future researchers who have the intention to develop a new population health index and to assess the suitability of the chosen method.

It is timely to conduct another review on population health indices given that Kaltenthaler et al’s systematic review was published more than a decade ago and we found no other systematic review conducted on population health indices since then. This study has two objectives, namely, (1) to provide an overview of existing population health indices in the literature, and (2) to examine the methods used to develop these population health indices.

## METHODS

### Approach

The scoping review methodology was chosen to identify the range of population health indices available from existing literature [11-14]. Arksey and O’Malley outlined five stages of a scoping review: identifying a research question; identifying relevant studies; selecting studies; charting the data; and collating, summarizing and reporting the results [11]. The exploratory nature of our research objective makes a systematic review method, which requires a focused question, not feasible; whereas, the breadth of a scoping review, which considers many different study designs to identify all relevant literature is more appropriate for this study [11,15]. After selecting the relevant studies, the population health indices identified were analysed to examine the methods used to develop these indices. The methodology paper by the Joanna Briggs Institute was used as a guide to conduct this scoping review [14]. Where appropriate, The Preferred Reporting Items for Systematic Reviews and Meta-Analyses (PRISMA) guided the reporting of this scoping review (Appendix A in **Online Supplementary Document**) [9].

### Eligibility criteria

Publications selection were guided by the ‘population’, ‘concept’ and ‘context’ (PCC) elements which ensure the coverage of a broader scope of study designs with less restrictive criteria [14,16]. No imposition was made for population criteria, since the objective of this scoping review is to give an overview of population health indices in general regardless of which population it focuses onto, for example, whether children or native population.

The first concept applied to guide the selection is ‘index’, where we used the concept of ‘composite indicator’ as defined by the OECD [4]. The second guiding concept is ‘health’, where the dimensions measured by the index are health outcomes, not a health determinant or a social determinant of health. Only publications with population health indices covering these two concepts are eligible for inclusion.

In the case of ‘context’, indices measuring a health concept at individual-level (eg, body mass index, patient satisfaction index, Cornell medical index) were excluded since the interest of this review is to search for population-level health indices. We decided not to use Kaltenthaler et al’s criterion, which excluded indices that combined only one health indicator with other non-health indicators to form an index because the inclusion of one health indicator qualifies the index to be considered as a health index. This criterion used in Kaltenthaler et al ended up excluding quality-adjusted life years (QALY), which, in our opinion, is important to be included because of its widespread use in population health measurement [17,18]. This scoping review did not limit to just original research journal articles but also included reports and journal review articles. No publication date or publication status restrictions were imposed. Only English language publications were considered.

### Scoping for population health indices

Publications were first identified by searching electronic databases. The search was applied to two biomedical databases (PubMed on 3rd March 2016 and CINAHL on 10th March 2016) and one multidisciplinary database (Emerald on 10th March 2016). The search strategy (Appendix S2 in **Online Supplementary Document**) was developed together by KA, CHT and CJN. We have combined the concept of ‘develop’ with ‘health’, ‘index’ and ‘context’ to narrow down to publications that also discussed the development process of their health indices. Supplementary search was conducted on 19^th^ May 2016 using Google search engine. All searches were conducted by KA with guidance from CJN. Bibliographic management software, EndNote X7, was used to import and manage the publications. The reference lists of the included publications were also scanned to further identify relevant publications.

### Screening publications

First, the publications identified were screened on the basis of title and abstract. We subsequently retrieved the full-text of the relevant publications to further assess their eligibility. The reference lists of all the eligible publications were examined to look for additional eligible publications. The screening process was conducted by KA and cross-checked with CJN and CHT regularly in batches.

### Charting the data

Data charting was conducted in two phases: (1) the included publications, and (2) the population health indices identified from the included publications. We developed a data charting map (Appendix C in **Online Supplementary Document**) according to the two objectives of this study to guide the charting process. The data charting map was pilot tested on seven randomly selected included publications, and refined accordingly.

### Collating

The collation was conducted in two parts. First, we sorted the identified population health indices according to the year they first appeared in the included publications. Brief description of the indices was made about its purpose, data, target population, metric and application. We further organized the indices into several categories based on the description made.

The second part observed the development process of these indices. We developed *a priori* analytical framework, which was adapted from the OECD’s ‘Handbook on Constructing Composite Indicators’ [19]. The simplified framework consists of four processes: (1) underpinning theory, model or framework; (2) data selection and processing; (3) formation of index; and (4) testing of index. All identified indices were mapped against these four processes of development.

## RESULTS

### Search results

**Figure 1:**
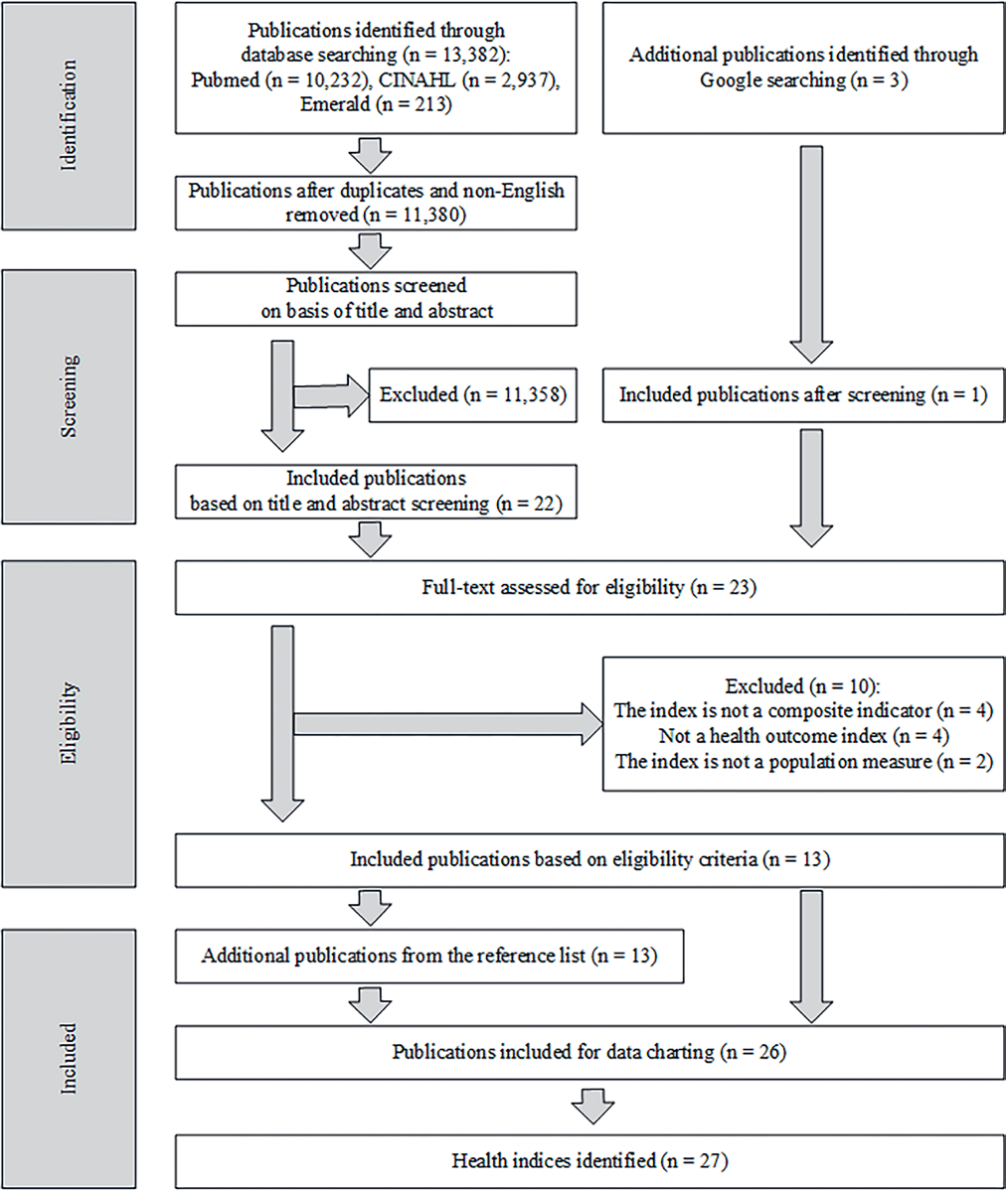
PRISMA flow diagram.

The electronic database search retrieved 13 382 publications (Figure 1). Twenty-six publications were included after screening through titles, abstracts and full texts.

### Characteristics of included publications and identified population health indices

Out of the 26 included publications, most were published as original research articles (65%) and half were published before year 2000. Twenty-seven population health indices were identified from the included publications (**Table 1**). The population health indices varied in their nomenclature; ‘index’ was the term most used (n = 16), followed by ‘indicator’ (n = 6) [24,33,45], ‘measure’ (n = 2) [29,34], ‘composite indicator’ (n = 2) [37], ‘composite index’ (n = 2) [38,40], and ‘ranking’ (n = 1) [44].

**Table 1:** Description of health indices identified from the scoping review

Name of index (reference)	Publication type (year)	Purpose of index	Data	Target population	Metric and application
Chiang’s H index [8,20]	Original article (1965), Review article (2004)	H index is a mathematical construction for describing the state of health of a well-defined population over a given period of time, such as a calendar year.	Monthly distribution of mortality for a given year; frequency of illness; duration of illness.	Hypothetical population.	H index is a mean duration of health in the range between 0 and 1; the healthier a population is, the larger will be the value of H.
Miller’s Q index [8,21]	Report (1970), review article (2004)	The Q index was developed as a tool to assist decision making with regards to program priorities by using the approach of management science. It combines mortality and morbidity in a single index to help distinguish the diseases that are amenable to treatment from those that are not.	Age and sex adjusted mortality for both target and reference population; crude mortality rate for target population; years of life lost due to premature death for target population; hospital days for target population; outpatient visits for target population.	North American Indian subpopulation in the United States of America.	Computed Q values was compared between 17 classes of disease according to the International Classification of Diseases. Higher computed Q value represent higher priorities.
Expectation of life free of disability [22] / Life expectancy free of disability (LEFD) [8,23] / Disability-free life expectancy (DFLE) [24-27]	Report (1971), original article (1983), original article (1996), review article (1996), original article (1997), review article (2004), review article (2012)	DFLE combines information of mortality and morbidity. It integrates disability and handicap data into the conventional life table models.	Number of deaths by age group; population size by age group; disability and handicap data.	General population of a country. The first report computes DFLE for United States of America.	Number of years a population expected to live free of disability.
K index [8,28]	Original article (1976), review article (2004)	The K index was developed to measure the quality of health care. It combines measure of incidence, severity and concentration of sentinel health events in communities.	Unnecessary mortality caused by specific conditions (number of deaths and age at death); unnecessary disability caused by specific conditions (number of disability days and concentration of severity)	Hypothetical community of 1000 people.	K scores in the range of the normative standards (the lowest score) to 1. The lowest score is the one with a health care system that has been most effective.
Quality-adjusted life years (QALY) [25,29]	Original article (1976), review article (2012)	QALY was developed to compare expected outcomes for a disease from different interventions. It is a health gap measures that combines duration of live and a measure of quality of life.	Utility function of health; probability of change in the utility function due to intervention	The first article employed a hypothetical utility function in the analysis.	QALY gained per 100 000 population. Comparison of QALY’s can be made among classification of diseases and interventions.
Gross national health product (GNHP) [8,30]	Original article (1979), review article (2004)	GNHP was developed to overcome the problem of incommensurability of measurement unit in the design of Linder’s gross national health deficit. It blends a nation’s mortality and disability statistics into a single number in units of disability-free life years per 100 000 population to compare the gross health status of regions.	Total number of deaths from all causes by age; total number of disability days from both acute and chronic conditions by age; size of population by age; life expectancies according to age	General population of United States of America.	Units of disability-free life years per 100 000 population. The highest GNHP value is considered the best in terms of overall health status. Comparing four regions in the United States of America (northeast, north central, south and west).
Chen’s G index [8,31]	Original article (1983) on the Canadian Indian health status index was used as reference since the original source of G index was unattainable. Review article (2004)	G index was developed based on Miller’s Q index (1970). It measures the health of disadvantaged minority groups in the United States such as the native American population.	Crude disease specific mortality for reference and target population; hospital days, clinic visits for disease specific morbidity.	Subpopulations of the United States of America (eg, native American).	The value of G range from zero, which indicates parity of health status, to some positive number, indicating the extent and severity of the disparity. If the target population fares better than the reference population, then G becomes negative. Comparison was made among type of diseases to assist decision making in resource allocation.
Canadian Indian health status index [8,31]	Original article (1983), Review article (2004)	The Canadian Indian health status index was developed to assist in health care resource allocation for preventive medicine program and to permit participation by the Indians for the selection of health program priorities. It was a revised version of Chen’s G index which was developed for the United States of America.	Age specific mortality; number of days of disease specific hospitalisation	Indians of Northern Ontario, Canada	Similar to the G index, the metric of Indian health status index represents the health status parity of the target population as compared to the reference population. Comparison was made among type of diseases to assist decision making in resource allocation.
General index of health [8,32]	Report (1992), review article (2004)	General index of health was developed as a tool to identify priority geographic area for the distribution of health resources among the residents of the city of Vancouver. It measures the general health of Vancouver population.	External causes of mortality (per 1000 population); mortality among 15 to 64 y old (per 1000 population); incidence of low birth weight (per 100 livebirths)	Residents of the city of Vancouver, Canada.	Index score is ranged between 0 to 30 points. It compares 12 geographic regions of the city of Vancouver.
Life expectancy free of avoidable mortality (LEFAM) [8,33]	Original article (1993), review article (2004)	LEFAM combines the concept of avoidable deaths with life expectancy. It measures the mean years an individual is expected to live if the health system is as efficient as it should be.	Number of deaths by age group coded with causes of death; population size by age group	General population of Spain (1983-1986).	Expected life free of avoidable mortality in years.
Disability-adjusted life years (DALY) [25,34,35]	Report (1993), review article (2012), original article (2012)	DALY was first introduced in the World Development Report 1993 to assess the global burden of diseases. It is a health gap population summary measure that combines years of healthy life lost due to disability with those that are lost from premature death.	Number of deaths; number of incident cases; life expectation or duration of disability; age at onset of disease.	General population according to diseases as classified by ICD.	Years lost per 1000 population per year. The comparison was made among the classification of diseases according to ICD.
Index of child mortality (ICM) [36]	Original article (1995)	ICM was developed for longitudinal assessment of health status of children. It combines five child mortality indicators.	Still birth rate; perinatal mortality rate; neonatal mortality rate; infant mortality rate; under five mortality rate	Child population in India (1972-1988)	Decreasing trend of ICM over the years depicts better outcome of child mortality over the years. It compares 15 states in India.
Handicap-free life expectancy (HFLE) [24,27]	Review article (1996), original article (1997)	HFLE summarises the expected number of years to be lived free of handicap.	Population size according to age group; number of deaths according to age group; prevalence of handicap according to age group	General population of a country.	Expected years to be lived free of handicap.
Disability-adjusted life expectancy (DALE) [25] / Health-adjusted life expectancy (HALE) [24,25]	Review article (1996), review article (2012)	DALE summarises the expected number of years to be lived in what might be termed the equivalent of full health.	Population size according to age group; number of deaths according to age group; prevalence of disability according to age group	General population of a country.	Expected years to be lived in full health.
Healthy life expectancy (HLE) [24,27]	Review article (1996), original article (1997)	HLE summarises the expected number of years to be lived in self-perceived good health.	Population size according to age group; number of deaths according to age group; number of years perceived in poor health	General population of a country.	Expected years to be lived in self-perceived good health.
Dementia-free life expectancy [24]	Review article (1996)	Dementia-free life expectancy summarises the expected number of years to be lived free of dementia.	Population size according to age group; number of deaths according to age group; prevalence of dementia according to age group	General population of a country.	Expected years to be lived free of dementia.
Healthy life years (HeaLY) [25,37]	Original article (1998), review article (2012)	HeaLY is a health gap population summary measure that combines the amount of years of healthy life lost due to death with those that are lost due to morbidity. It was developed to assess the effects of health interventions, some of which may have impact on more than one disease.	Incidence rate per 1000 population per year; average age at onset; average age at death; expectation of life at age of onset; expectation of life at age of death; case fatality ratio; case disability ratio; extent of disability; duration of disability in years.	General population of a country. The first article computes HeaLY using the Ghana national data set.	Healthy life years lost per 1000 population per year. Comparison was made according to diseases as classified by the ICD.
Townsend’s overall health index [8]	Review article (2004)	Overall health index was developed to compare the health of population in small areas in Britain. The index was also used to compare with Townsend’s deprivation index. Mortality, sickness and low birth weight data was combined to form the index.	Standardised mortality ratios of population below 65 y (premature mortality); proportion of all residents who classified themselves as permanently sick or disabled; proportion of live births under 2.8kg (low birth weight).	Specified area general population.	The comparison was made among electoral wards in United Kingdom.
Plymouth health district index [8]	Review article (2004)	Adaptation of Townsend’s overall health index to be used in Plymouth health district, United Kingdom.	Standardised mortality ratio; infant mortality ratio; proportion of residents in private households classified as permanently sick or disabled	District’s general population	The comparison was at district level comparing the health status of electoral wards.
Index of multiple deprivation [8]	Review article (2004)	Index of multiple deprivation was used to compare electoral wards with regards to deprivation and health. It measures health deprivation at electoral ward level.	Comparative mortality ratios for men and women at ages under 65 y; people receiving attendance allowance or disability living allowance as a proportion of all people; proportion of people of working age receiving incapacity benefit or severe disablement allowance; age and sex standardised ratio of limiting long-term illness; proportion of low birth weight (<2.5kg).	General population at electoral ward level.	The comparison was made among electoral wards in United Kingdom.
Child health index [38]	Original article (2005)	The child health index was developed using data from Kids Count Data Book 2002 [39]. The index was developed to construct a composite health index that would facilitate statistical analysis by state for overall physical health of children, because each Annie E. Casey Foundation (AECF) indicator reflects only a single health dimension. It measures overall physical health of children.	Percent of low birth weight infants; infant mortality rates; child death rates; teen birth rates.	Child population in United States of America.	The range of scores falls between -3 and 3. Scores closer to 3 represent better health, and those closer to -3 represent poorer health. States were ranked in order of best to worst on the basis of the composite score (Rank 1: the score closest to 3). It is a national comparison covering 50 states in the United States of America.
Composite index of anthropometric failure (CIAF) [40]	Original article (2005)	The CIAF provides an overall estimate of undernourished children in a population using a composite measure, in the argument that standard indices of stunting, wasting and underweight may each be underestimating the scale of undernutrition problem. It adapted Svedberg’s model of anthropometric failure as an aggregate measure of undernutrition that identifies all undernourished children due to wasting, stunting or underweight [41].	Prevalence of stunting among children; prevalence of wasting among children; prevalence of underweight among children.	Children in India.	CIAF scores is presented in the range of 0 to 100 (in percentage).
Global nutritional index (GNI) [42]	Original article (2008)	The GNI was developed to assess a nation's overall nutrition status, and not just hunger (both nutritional deficiency and excess). It measures overall nutritional status constructed from estimates of nutritional deficits, excess and food security.	Age-standardized DALYs lost due to nutritional factors; Percentage of women age 15 to 100 with BMI greater than or equal 30; Percentage of the population with undernourishment.	General population of a country.	GNI scores are presented in the range of 0 to 1, with higher scores indicating better nutrition status. It is a cross-country comparison covering 192 countries. For GNI, the countries were divided into four groups: developed countries, countries in transition, low-mortality developing countries, and high-mortality developing countries. Whereas, GNIg represent the ranking of all countries worldwide and not by their development.
Inequity-in-health index (IHI) [43]	Original article (2008)	IHI is a bi-dimensional composite index allowing inequity in health to be quantitatively estimated and graphically represented in countries, regions and around the world. It measures health inequity in countries assuming inequity as “inequality of health outcomes”.	Underweight children; child mortality; death from malaria among children aged 0-4; death from malaria at all ages; births attended by skilled health personnel; immunization against measles.	General population of a country.	IHI scores is presented in the range of 0 Pi to 1 Pi, with higher area scores indicating higher inequality of health outcomes. Ranking was made according to country area scores. Country with lowest area score ranked number 1, whereas the highest area score ranked number 127. It is a cross-country comparison covering 127 countries.
Wisconsin county health rankings [44]	Report (2008)	The Wisconsin county health rankings was developed to encourage discussion about important population health issues among Wisconsin public health and other policy communities. It measures the health level of each county (population health).	Years of potential life lost; self-reported general health status.	Counties general population.	Ranked by overall health outcomes; county ranked number 1 scored the best overall health outcomes. It covers 72 counties in Wisconsin state.
Mortality_ABC_ index [45]	Original article (2014)	The index was developed to add to the inequality debate in the health domain. It measures absolute mortality (A), mortality inequality (B) and mortality clustering (C).	Infant mortality rate; Theil index; G statistic.	General population of a country.	Each country is ranked in terms of permutations of its three-part source (A, B, C). It compares 130 countries.
The EIU outcomes index [46]	Report (2014)	The EIU Outcomes index was developed to measure health outcomes of countries. The outcomes were compared with spending to assess value for money in health care.	Disability-adjusted life years; Health-adjusted life years; Adult mortality rates; Life expectancy at age 60.	General population of a country.	The EIU Outcomes index scores range from 0 to 100, with higher scores indicating better outcomes. It compares 166 countries.

### Index topics

Fifteen of the indices measure overall health outcomes (eg, Chiang’s H index, gross national health product) [8,20,22,24,30,33,36,38,44,46], four indices measure specific health outcomes (eg, undernourishment [40], nutrition [42], inequality of mortality [45], and inequality of health outcomes based on Millennium Development Goals [43]) and six indices measure population health based on disease-specific outcome (eg, Miller’s Q index, Chen’s G index) [21,29,31,34]. Two indices focused on the topic of quality: ‘K index’ on the quality of health care [28], and ‘quality-adjusted life years’ on quality of health [28,29]. Four indices were developed as a measure to assist decision making in health resource allocation (eg, Canadian Indian health status index, general index of health) [21,31,32].

### Target population

Twenty-one of the population health indices measure health for general populations; three measure hypothetical populations [20,28,29], while the other 18 measure real populations [8,22,24,30,32-34,37,42-46]. Thirteen indices cover country-level populations [22,24,30,33,34,37,42,43,45,46], while five indices cover smaller areas (ie, electoral wards, districts and counties) [8,32,44]. The remaining six indices measure defined subpopulations; three cover children [36,38,40], and another three indices target specific ethnic or native population [21,31].

### Index data

Out of 27 population health indices, all used mortality data except one (composite index of anthropometric failure [40]). Five of the indices coupled mortality data together with birth data [8,32,36,38]. Morbidity data were used in two ways to represent health status: (1) using disability or handicap data in general [8,20,21,24,28,30]; (2) using disease-specific morbidity data [24,34,37,40,42,43]. Other categories of indices include: five indices used data of health care service delivery or intervention [21,29,31,43]; five used physical well-being data [8,24,29,44]; index of multiple deprivation used welfare data to form the index [8]; nine indices used general population characteristics (eg, population size by age group) and life expectancy data [22,24,30,33,42,46].

#### Development processes

Fourteen population health indices reported at least one of the development processes in the simplified framework (**Table 2**). Seven indices reported use of a theory, model or framework to provide a basis for combining individual indicators into a meaningful index [36,37,40,42-45]. Only eight indices explained how the data was chosen or processed [30,36,38,40,42,43,45,46]. Twelve indices reported the methods used to form the index [8,21,30,32,33,36-38,40,42,43,45]. Only nine indices were tested statistically after they were formed [21,33,36-38,40,42,43,46].

**Table 2:** Health indices by the processes of development

Name of index	Theory, model or framework	Data selection and processing	Formation of index	Testing of index
Miller’s Q index [8,21]	Not reported.	Health data by classes of disease from the Publication Health Services (PHS) publication was used. Mortality rates of both reference and target population were adjusted for age and sex.	The Q index is formed using a mathematical formula.	Diseases ranked by Q were compared (correlation) with the individual data of deaths, inpatients and outpatients that was originally used to compute the Q values.
Gross national health product (GNHP) [8,30]	Not reported.	Data was obtained from two the National Center for Health Statistics (NCHS) publications that provide life-table and disability data for 1971. Population size by age is available in NCHS computer printouts used in computing mortality rates by age. The age group were collapsed to achieve comparability because the two NCHS publication displayed mortality and disability data by different age groups.	Gross national health product was calculated using a mathematical formula.	Not reported.
General index of health [8,32]	Not reported.	Not reported.	Each component is assigned a score between 0 to 10 points. The region with the lowest incidence is assigned 10 points, the highest incidence 0 points and the other regions are ordered by deciles. Scores of each component are then added to have the general index of health with a range between 0 to 30 points.	Not reported.
Life expectancy free of avoidable mortality (LEFAM) [8,33]	Not reported.	Not reported.	Using life table calculation with additional calculation: Subtracting the number of avoidable deaths from the total deaths according to age.	LEFAM is compared with life expectancies by age group. LEFAM and life expectancies is correlated with several variables: mortality rate, infant mortality rate, gross domestic product, hospital beds, health human resources, ambulatory consultations.
Index of child mortality (ICM) [36]	Improved upon the methodological approach to compute an index of health developed by Chandra Sekhar et al. (1991)	Data was assessed for its completeness according to states and years.	Composition of index using factor analysis. The score for ICM is the sumproduct of factor scores and percentage of variation explained by each factor.	Comparison between ICM and under five mortality rate (U5MR).
Healthy life years (HeaLY) [25,37]	Uses the pathogenesis and natural history of disease as the conceptual framework for assessing morbidity and mortality for interpreting the effects of various interventions	Not reported.	Using mathematical formula.	Compare with DALY.
Index of multiple deprivation [8]	Not reported.	Not reported.	'Shrinkage' procedure applied to all data, factor analysis to generate weights to combine indicators, index is ranked then domain standardised and transformed to an exponential distribution; individual domains are weighted (health is 15%) and combined to produced ward index score	Not reported.
Child health index [38]	Not reported.	Indicators were chosen on the basis of historic and routine use to define health outcomes in children and their inclusion as objectives for Healthy People 2010.	Normalisation: Indicators were calculated as rates or percentages. Standard scores were calculated for each state for each health indicator by subtracting the mean of the measures for all states from the observed measure for each state. The resulting measure was then divided by the standard deviation (SD) and multiplied by -1. Weighting: All measures were given the same weight in calculating the overall standard score. Aggregation: Summation for all standard score of each indicator.	Decomposition: Illustrates the differences between the Deep South and other combined regions for each of the health indicators. Link to others: Multivariate analysis was done with variables that was decided to be potential confounders not health outcomes for children.
Composite index of anthropometric failure (CIAF) [40]	Adaptation from Svedberg's framework of anthropometric failure which identified six group of children.	Children with grossly improbable z-scores of anthropometric failure were flagged and excluded.	The CIAF excludes those children not in anthropometric failure and counts all children who have wasting, stunting, or are underweight.	Analysis of variance was used to examine the relationship between undernutrition and standard of living; age-adjusted logistic regressions were used to examine the relationship between undernutrition and morbidity. Children who were not in anthropometric failure (ie, group A) are set as the reference group in each analysis.
Global nutritional index (GNI) [42]	Discuss overall requirements for a well-nourished person, constructed from estimates of nutritional deficits, excess, and food security.	Data were chosen on the basis of comprehensiveness (measuring all aspects of the area), completeness (availability for all countries), and comparability (the appropriateness of comparisons of the measure among countries).	Normalisation: Three indicators were normalised into 0 to 1 scale. Weighting: Because of the lack of an obvious or evidence-based way to weight the three parameters of nutrition, it was decided to weight them equally, as in the HDI. Aggregation: Average value of the three indicators were subtracted from 1 to invert the scale. Because in each indicator a higher value indicated a worse outcome. This invertion made the final score between 0 and 1, with higher scores indicating better nutrition status.	Correlation made between HDI and GNIg.
Inequity-in-health index (IHI) [43]	Developing the IHI using indicators proposed for monitoring progress of the MDG. The index is bi-dimensional composite: estimating inequity in health quantitatively and representing it graphically.	Data selection: (1) Variables were selected if they were individually registered in more than 40% of total countries more than 90 countries). (2) Social determinants of health were not included because we assume the point of view of health outcomes to measure inequity in health. Data exploration: (1) Disparity of countries data were explored using median and Attributable Fraction (AF). (2) Variables were excluded if high uniqueness was verified in at least two factor analysis methods (ie, iterated main factor, maximum likelihood factor method, or main component factor method). Initially, 14 variables was selected, in the end only 6 variables remain.	Normalisation: Attributable Fractions allow relative differences between countries to be estimated. Composition: The scores from the two factors for each variable were obtained by main component analysis and plotted as x and y-axis on a Cartesian plane. Each variable’s area was then calculated as a product of both axes. The sum of all variance was represented as being a circle (360 degree). The percentage of each variable’s area was calculated with respect to the sum of all variable areas. In terms of angles, each vector’s size was the fraction attributed to a specific country in a specific outcome. Each variable therefore had two components represented in the circle: the bi-dimensional score of its variance (angle) and the size of its disparity in health, compared to the best country (attributed fraction vector).	Reliability: Reliability analysis between the three different method getting the area score. Validity: Validity of the index was done by finding linkage with other indicators: Human development index, health gap indicator, human poverty index, life expectancy, and the probability of dying before 40 y of age. Discriminant validity: Discriminate countries by income, region, corruption and level of indebtedness
Wisconsin county health rankings [44]	Adaptation from Kindig and Stoddart population health framework.	Not reported.	Not reported.	Not reported.
Mortality_ABC_ index [45]	Draw upon the literature of population health and public health to develop a multidimensional measures covering 3 components of mortality (absolute mortality level, mortality inequality, mortality clustering)	Data was selected according to availability and timeliness.	Comparability: Absolute mortality level (A) and mortality inequality (B) were grouped in tertial classifications (high, medium, low). Mortality clustering (B) were classify into 2 groups: spatial autocorrelation present within the country (significant and not significant). Composition: All the ABC score were paired in three to get the distribution of countries in three-part mortality indicator	Not reported.
EIU outcomes index [46]	Not reported.	DALYs and HALEs was chosen due to expediency. Adult mortality rates and life expectancy at age 60 were added as extra measures since both DALYs and HALE weight young people and children more heavily than older ones.	Not reported.	Compares the outcomes score with spending in health.

## DISCUSSION

This scoping review provides an updated overview of existing population health indices in the literature and examines systematically the methods used in developing these indices. Existing reviews mainly cover indices that originated from prominent international organizations. Van de Water et al produced an inventory of indices that originated from European Union member states [24], while Hyder et al presented a narrative review of indices that originated from the World Bank and the World Health Organization [25]. Our review, which also covers indices that were born out of research practice, revisits Kalthenthaler et al.’s attempt in conducting a systematic search of published indices in the literature. The method we used included 26 publications and identified 27 health indices documented in the period that spans 50 years (1965-2014).

The included population health indices highlights that most health indices focus on a population’s overall health outcomes, or the health of a general population. Measuring health with a more focused purpose or on a specific subpopulation allows for specific policy or intervention to be developed and implemented. For instance, the composite index of anthropometric failure (CIAF) estimates the number of undernourished children in a population. It demonstrates that the prevalence of undernutrition among children in India was greatly underestimated by the using standard indicators of stunting, wasting or underweight [40]. Global nutritional index (GNI) highlights the trade-off between the problem of undernutrition and obesity at a global scale that may assist policy decision making in global nutrition aid [42]. This scoping review only managed to identify three indices that measure outcomes of children’s health and another three that measure the health outcomes of specific ethnic or native population. Similarly, women’s health issues have received relatively more social interest compared to men’s health issues, despite data have been showing men are faring worse in various aspects of health [47,48]. A population index that measures various outcomes of men’s health in a single index, and allows for cross-country comparison, may provide the needed exposure of this problem more strategically.

The usage of a theory, model or framework as the basis to develop measures of health outcomes is lacking but necessary [49]. It helps readers to understand better by providing the context into the multidimensional concept of the index. It identifies whether the indicators used is a measure of health outcomes or health determinants, and provides insight into the relationships between indicators. The inclusion of a theory, model or framework will ensure a population health index is not merely a statistical amalgamation of indicators but also founded on valid health concepts.

### Limitations

Our search identified 13 publications from the eligibility criteria. The search is ostensibly narrow, but the outcome is due to two concepts that we used in our search strategy: ‘INDEX’ and ‘DEVELOP’ (Appendix B in **Online Supplementary Document**). Publications that defined their population health measure as ‘INDICATOR’ are omitted in our initial search since indicators are components of an index, and they do not present the full reflection expected of an index. Since our second objective is to examine the methods used to develop these population health indices, ‘DEVELOP’ was included as a concept in the search. Publications on population health indices with titles and abstracts that do not fulfil this concept are further excluded in the initial search.

A supplementary search using Google search engine was conducted. However, specific website search, for instance search on population health indices in the websites of relevant government departments, was not conducted.

Our review also did not use the Peer Review of Electronic Search Strategies (PRESS) to help guide and improve the peer review of our electronic search strategies, as PRESS was published after the completion of the search.

Although scoping review methodology does not require appraisal of the study [11-13,50], a quality assessment of population health indices is important. Currently there is no guideline to allow critical comparisons of existing indices. Development of such a guideline will enable comprehensive assessment of the quality of health indices which is essential to advance the methodological discourse for developing population indices in health research.

Our review only includes indices that measure a multidimensional health outcome. This review excluded indices that measure solely determinants of health (eg, Urban Health Index) or well-being of a population (eg, Human Development Index, OECD Better Life Index). Future reviews may want to include other indices that cover non-health outcomes.

## CONCLUSION

Even after 50 years of ongoing usage of population health indices, knowledge in this area are rarely discussed in academic literature. This scoping review highlights two important gaps. First, most of the population health indices measure a population’s overall health outcomes, but only few gave focus to specific health topics or health of specific sub-populations. Second, there is a lack in the usage of theory, model or framework as a basis to develop health concept measurement. This process is important as it ensures the overall validity of the developed population health index. A more in-depth analysis of population health indices is required so that methodological developments and refinements are continuous in this area of knowledge. It is necessary to develop a guideline on how population health indices can be developed systematically and rigorously.

## Additional Material

Online Supplementary Document
