# Supplementary material for: Population indices measuring health outcomes: A scoping review
Source: J Glob Health. 2019 Jan 23;9(1):010405. doi: 10.7189/jogh.09.010405 (PMC6344069; doi:10.7189/jogh.09.010405)
Supplement: Online Supplementary Document [file jogh-09-010405-s001.pdf]

J Glob Health 2019; 9: 010405

Appendix S1: PRISMA checklist to guide the reporting of the scoping review

Section/topic	#	Checklist item	Reported on page #
**TITLE**			
Title	1	Identify the report as a systematic review, meta-analysis, or both.<br>*Remarks: A scoping review*	1
**ABSTRACT**			
Structured summary	2	Provide a structured summary including, as applicable: background; objectives; data sources; study eligibility criteria, participants, and interventions; study appraisal and synthesis methods; results; limitations; conclusions and implications of key findings; systematic review registration number.	Abstract
**INTRODUCTION**			
Rationale	3	Describe the rationale for the review in the context of what is already known.	2
Objectives	4	Provide an explicit statement of questions being addressed with reference to participants, interventions, comparisons, outcomes, and study design (PICOS).	2
**METHODS**			
Protocol and registration	5	Indicate if a review protocol exists, if and where it can be accessed (e.g., Web address), and, if available, provide registration information including registration number.	NA
Eligibility criteria	6	Specify study characteristics (e.g., PICOS, length of follow-up) and report characteristics (e.g., years considered, language, publication status) used as criteria for eligibility, giving rationale.<br>*Remarks: We used PCC*	3
Information sources	7	Describe all information sources (e.g., databases with dates of coverage, contact with study authors to identify additional studies) in the search and date last searched.	3

Section/topic	#	Checklist item	Reported on page #
Search	8	Present full electronic search strategy for at least one database, including any limits used, such that it could be repeated.	Appendix B
Study selection	9	State the process for selecting studies (i.e., screening, eligibility, included in systematic review, and, if applicable, included in the meta-analysis).	3
Data collection process	10	Describe method of data extraction from reports (e.g., piloted forms, independently, in duplicate) and any processes for obtaining and confirming data from investigators.	4
Data items	11	List and define all variables for which data were sought (e.g., PICOS, funding sources) and any assumptions and simplifications made.<br>*Remarks: Data charting map*	Appendix C
Risk of bias in individual studies	12	Describe methods used for assessing risk of bias of individual studies (including specification of whether this was done at the study or outcome level), and how this information is to be used in any data synthesis.	NA
Summary measures	13	State the principal summary measures (e.g., risk ratio, difference in means).	NA
Synthesis of results	14	Describe the methods of handling data and combining results of studies, if done, including measures of consistency (e.g., I^2) for each meta-analysis.	NA
Risk of bias across studies	15	Specify any assessment of risk of bias that may affect the cumulative evidence (e.g., publication bias, selective reporting within studies).	NA
Additional analyses	16	Describe methods of additional analyses (e.g., sensitivity or subgroup analyses, meta-regression), if done, indicating which were pre-specified.	4
**RESULTS**			
Study selection	17	Give numbers of studies screened, assessed for eligibility, and included in the review, with reasons for exclusions at each stage, ideally with a flow diagram.<br>*Remarks: Flow diagram presented in Figure 1*	4, 8
Study characteristics	18	For each study, present characteristics for which data were extracted (e.g., study size, PICOS, follow-up period) and provide the citations.	4, 5

Section/topic	#	Checklist item	Reported on page #
Risk of bias within studies	19	Present data on risk of bias of each study and, if available, any outcome-level assessment (see Item 12).	NA
Results of individual studies	20	For all outcomes considered (benefits or harms), present, for each study: (a) simple summary data for each intervention group and (b) effect estimates and confidence intervals, ideally with a forest plot.	NA
Synthesis of results	21	Present results of each meta-analysis done, including confidence intervals and measures of consistency.	NA
Risk of bias across studies	22	Present results of any assessment of risk of bias across studies (see Item 15).	NA
Additional analysis	23	Give results of additional analyses, if done (e.g., sensitivity or subgroup analyses, meta-regression [see Item 16]).<br>*Remarks: Presented in Table 2*	5, 19
**DISCUSSION**			
Summary of evidence	24	Summarize the main findings including the strength of evidence for each main outcome; consider their relevance to key groups (e.g., health care providers, users, and policy makers).	5, 6
Limitations	25	Discuss limitations at study and outcome level (e.g., risk of bias), and at review level (e.g., incomplete retrieval of identified research, reporting bias).	6, 7
Conclusions	26	Provide a general interpretation of the results in the context of other evidence, and implications for future research.	7
**FUNDING**			
Funding	27	Describe sources of funding for the systematic review and other support (e.g., supply of data); role of funders for the systematic review.	7

Appendix S2:Search strategy used for the three electronic databases

1. PubMed
Searched: 3rd March 2016
Records identified: 10,232

'HEALTH'
#1 health[tiab] OR "Health"[Mesh]

'INDEX'
#2 Index[tiab] OR indices[tiab] OR indexes[tiab] OR ranking[tiab] OR "league table"[tiab] OR "league tables"[tiab] OR metric*[tiab] OR "report card"[tiab] OR "report cards"[tiab] OR "composite indicator"[tiab] OR "composite indicators"[tiab]

'CONTEXT'
#3 Global[tiab] OR nation[tiab] OR nations[tiab] OR national[tiab] OR "Developing Countries"[Mesh] OR "Developed Countries"[Mesh] OR world[tiab] OR country[tiab] OR countries[tiab] OR region[tiab] OR regions[tiab] OR regional[tiab] OR district[tiab] OR districts[tiab] OR international[tiab] OR geographic[tiab] OR geographics[tiab] OR geographical[tiab] OR population[tiab]

'DEVELOP'
#4 develop[tiab] OR develops[tiab] OR developed[tiab] OR development[tiab] OR construct[tiab] OR constructs[tiab] OR builds[tiab] OR build[tiab] OR built[tiab]

#5 - #1 and #2 and #3 and #4

2. CINAHL
Searched: 10th March 2016
Records identified: 2,937

'HEALTH'
S1 - (MH Health OR TI health OR AB health)

'INDEX'
S2 - (MH "Health Status Indicators" OR TI(index OR indices OR indexes OR ranking OR "league table" OR "league tables" OR metric* OR "report card" OR "report cards" OR "composite indicator" OR "composite indicators") OR AB(index OR indices OR indexes OR ranking OR "league table" OR "league tables" OR metric* OR "report card" OR "report cards" OR "composite indicator" OR "composite indicators"))

‘CONTEXT’
S3 - (MH(population OR “international agencies” OR “joint commission" OR "Government Agencies") OR TI(global OR nation OR nations OR national OR “developing countries” OR “developed countries” OR world OR country OR countries OR region OR regions OR regional OR district OR districts OR international OR geographic OR geographics OR geographical OR population) OR AB(global OR nation OR nations OR national OR “developing countries” OR “developed countries” OR world OR country OR countries OR region OR regions OR regional OR district OR districts OR international OR geographic OR geographics OR geographical OR population))

‘DEVELOP’
S4 - (TI(develop OR develops OR developed OR development OR construct OR constructs OR builds OR build OR built) OR AB((develop OR develops OR developed OR development OR construct OR constructs OR builds OR build OR built))

S5 - S1 AND S2 AND S3 AND S4

3. Emerald
Searched: 10th March 2016
Records identified: 213

HEALTH
S1 - [Abstract: health]

INDEX
S2 - [Abstract: index] OR [Abstract: indices] OR [Abstract: indexes] OR [Abstract: ranking] OR [Abstract: “league table”] OR [Abstract: “league tables”] OR [Abstract: metric*] OR [Abstract: “report card”] OR [Abstract: “report cards”] OR [Abstract: “composite indicator”] OR [Abstract: “composite indicators”]

CONTEXT
S3 - [Abstract: global] OR [Abstract: nation] OR [Abstract: nations] OR [Abstract: national] OR [Abstract: “developing countries”] OR [Abstract: “developed countries”] OR [Abstract: world] OR [Abstract: country] OR [Abstract: countries] OR [Abstract: region] OR [Abstract: regions] OR [Abstract: regional] OR [Abstract: district] OR [Abstract: districts] OR [Abstract: international] OR [Abstract: geographic] OR [Abstract: geographics] OR [Abstract: geographical] OR [Abstract: population]

DEVELOP

S4 - [Abstract: develop] OR [Abstract: develops] OR [Abstract: developed] OR [Abstract: development] OR [Abstract: construct] OR [Abstract: constructs] OR [Abstract: builds] OR [Abstract: build] OR [Abstract: built]

S5 – S1 AND S2 AND S3 AND S4

Appendix C: Data Charting Map

Publication information	Description of population health indices (Objective 1)	Development methods of population health indices (Objective 2)
1. Title<br>2. Authors<br>3. Year of publication<br>4. Publication type (original article, review article, report)	1. Name of index<br>2. Purpose of index<br>3. Data<br>4. Target<br>5. Metric and application	1. Framework<br>2. Data selection and processing<br>3. Formation of index<br>4. Testing of index
